# Hemorrhagic Pericardial Effusion From Apixaban Use: Case Report and Literature Review

**DOI:** 10.7759/cureus.30021

**Published:** 2022-10-07

**Authors:** Syed Alishan Nasir, Nishan Babu Pokhrel, Alyza Baig

**Affiliations:** 1 Internal Medicine, Norwalk Hospital, Norwalk, USA

**Keywords:** pericardial effusion. cardiac tamponade, hemopericardium, oral anticoagulation, non valvular atrial fibrillation, direct oral anticoagulant therapy

## Abstract

Direct oral anticoagulants (DOACs) have revolutionized therapy for stroke prophylaxis in patients with non-valvular atrial fibrillation. These medications are generally well tolerated and are not associated with the inconvenience of repeat international normalized ratio (INR) checks. While bleeding in general is a common side effect associated with DOACs, especially from a gastrointestinal source, spontaneous hemorrhagic pericardial effusions are not seen frequently. Herein, we present a case of a patient who developed a hemorrhagic pericardial effusion three days after the initiation of apixaban. We also summarize the current data that is available showing this side effect and highlight an important risk factor that may predispose patients to this complication.

## Introduction

Hemopericardium is usually caused by trauma, tumor or myocardial infarction [1]. Spontaneous hemopericardium with the use of direct oral anticoagulants [2,3], warfarin [4] and anticancer drugs like ibrutinib [5] is an infrequent occurrence. Anticoagulants implicated in hemopericardium are direct oral anticoagulants (DOACs) which include direct thrombin inhibitors (dabigatran) and direct factor X inhibitors (apixaban, rivaroxaban, edoxaban, betrixaban). Even though DOACs have a lower bleeding risk profile compared to warfarin, these are directly associated with a high risk of gastrointestinal bleeding, especially when using rivaroxaban, dabigatran and edoxaban [6]. Dabigatran was the first medication to be approved in 2010 [7]. Apixaban was approved in 2012 for the prevention of embolic events in patients with non-valvular atrial fibrillation and has since been approved for several other indications [8]. Apixaban reversibly inhibits free factor Xa and has a half-life of eight to 15 hours [9]. It has been the second most frequently used anticoagulation next to rivaroxaban, with the least used anticoagulant being edoxaban [10]. Herein, we present a case of a 93-year-old female with chronic lymphocytic leukemia (CLL), in remission with ibrutinib maintenance therapy, who was diagnosed with new-onset atrial fibrillation and who developed spontaneous hemopericardium following initiation of anticoagulation with apixaban. Investigations for possible pericarditis did not reveal abnormalities, and ultimately, the diagnosis of ibrutinib/apixaban-induced hemopericardium was made. She underwent pericardiocentesis and was ultimately discharged off both agents.

## Case presentation

**Figure 1:**
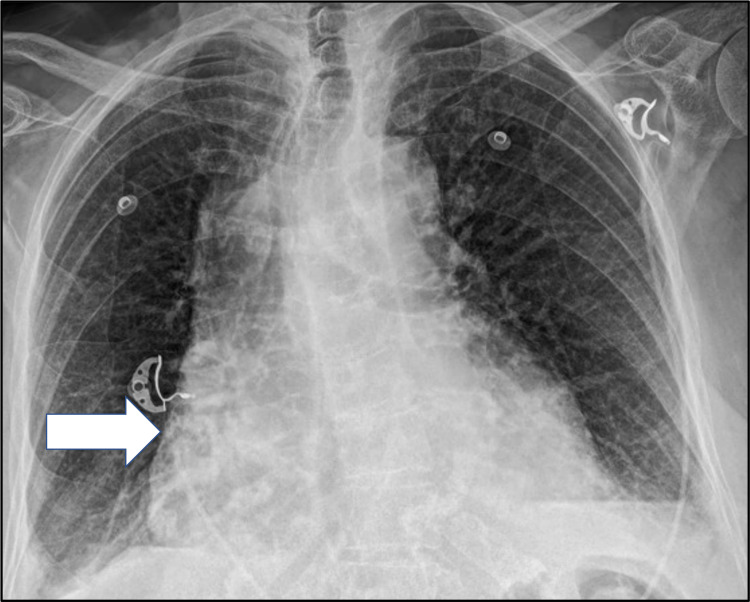
Chest x-ray showing cardiomegaly (white arrow)

A 93-year-old female with a past medical history of chronic lymphocytic leukemia (CLL) in remission, on treatment with ibrutinib, presented to her cardiologist’s office on August 26, 2021, with a weeklong history of palpitations, and was diagnosed with atrial fibrillation (a-fib) with rapid ventricular response (RVR). The patient was started on anticoagulation with apixaban 5 mg twice a day (BID) and was rate controlled with oral metoprolol 25 mg BID. Over the next three days, the patient continued to have worsening palpitations associated with episodes of lightheadedness and new-onset shortness of breath which prompted her to visit the emergency department (ED) at Norwalk Hospital on August 29, 2021. On initial evaluation, the patient was noted to be hypotensive with systolic blood pressures (SBPs) in the 80s and tachycardic with heart rates (HRs) in the 140-150s. EKG showed evidence of supraventricular tachycardia in the setting of atrial fibrillation with RVR without any acute ischemic changes. Laboratory studies were significant for normocytic anemia with hemoglobin (Hgb) of 10.2 g/dL and negative cardiac troponins. Chest X-ray showed cardiomegaly (Figure 1).

**Figure 2:**
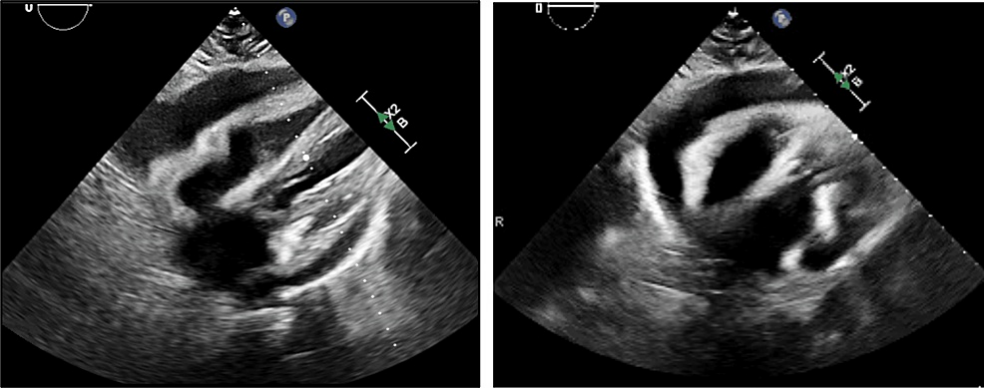
Transthoracic echocardiogram, two views, showing evidence of massive pericardial effusion along the right heart border

Given the hemodynamic instability in the setting of supraventricular tachycardia, the patient emergently underwent synchronized cardioversion resulting in conversion to normal sinus rhythm (NSR) and she was started on an amiodarone infusion while in the ED. Following cardioversion, her hemodynamics were noted to improve. An urgent echocardiogram was performed which revealed no new wall motion abnormalities; however, it was notable for a large pericardial effusion along the right ventricular free wall and right atrial free wall, with evidence of possible tamponade physiology (Figure 2).

Our patient underwent pericardiocentesis emergently the same day with the removal of approximately 450 cc of grossly hemorrhagic fluid. The pericardial catheter was left in place to drain any residual or additional fluid accumulation; another 300 cc of grossly bloody fluid was removed. IV amiodarone was switched to oral (PO). Fluid analysis was consistent with red, opaque fluid with inflammatory cells and red blood cells (RBCs). Pericardial fluid gram stain, bacterial culture and acid-fast bacilli culture were negative. Pericardial fluid cytology showed neutrophilic predominance but no obvious evidence of malignancy that may have contributed to the patient's pericardial effusion. Given the gross evidence of hemorrhagic pericardial effusion and recent initiation of anticoagulation, the differential for her large effusion included apixaban-induced hemorrhagic pericardial effusion. We also considered viral pericarditis and autoimmune pericarditis. Subsequent studies were remarkable for a negative anti-nuclear antibody (ANA), negative anti-histone antibody and negative IgM titers for cytomegalovirus (CMV), Epstein-Barr virus (EBV) and Coxsackie virus. A viral panel was negative for metapneumovirus, coronavirus, rhinovirus, parainfluenza virus, influenza virus, respiratory syncytial virus and adenovirus.

Following the pericardiocentesis, her apixaban was discontinued. Hematology/Oncology consultation was obtained, and a decision was made to discontinue her ibrutinib as well. Our patient’s drain was removed over the next few days, and a repeat echocardiogram showed no reaccumulation of pericardial effusion. Our patient continued to do well and was discharged off anticoagulation. She was followed in the outpatient setting by her cardiologist and underwent a repeat echocardiogram in June 2022 which showed no reaccumulation of pericardial effusion.

## Discussion

Hemorrhagic pericardial effusion remains an infrequent complication of DOAC use, and based on our literature review, patients who develop this generally present with evidence of heart failure and tamponade physiology which manifests as dyspnea and bilateral lower extremity swelling. Our patient presented with palpitations and episodes of lightheadedness that are usually less common presentations; however, this could be explained by the fact that she was in a state of unstable supraventricular tachycardia. Sheikh et al. [10] in their systematic review of 41 case reports found shortness of breath as the most common presentation (75.2%) followed by chest pain (57.2%) in patients who developed hemopericardium in the setting of DOAC use. Most of the patients were hemodynamically unstable on presentation, similar to ours. Lightheadedness, as in the case of our patient, has only been reported in three case reports [11-13]. Based on our literature review, there appears to be a temporal correlation between the onset of symptoms and the timing of anticoagulation initiation. Patients generally become symptomatic within two to 12 weeks after the initiation of DOACs, although there were some cases in which symptoms only presented after months of treatment [10].

While there have been reports showing evidence of hemopericardium spontaneously emerging from DOAC use, the concomitant use of ibrutinib appears to be linked to this adverse effect as well. Independently, the risk of a major bleeding event with ibrutinib has been reported to range from 7.6% to 19% in the literature [14,15]. In a retrospective analysis of 70 patients in the real-world scenario [15], major bleeding with ibrutinib occurred in 19% of patients. Baseline anemia (hemoglobin less than 12 g/dL), elevated international normalized ratio ((INR) more than 1.5), concurrent antiplatelets (70%) and anticoagulant use (17%) significantly increased the risk of major bleeding (grade 3 or more bleeding event). Hemorrhagic pericardial effusion requiring pericardiocentesis is a category 4 bleeding event [15] which occurred in our patient. Our patient also had mild normocytic anemia on presentation (hemoglobin of 10.2 g/dL) and was under anticoagulation with apixaban, both of which were potential risk factors to predispose our patient for a bleeding risk due to her ibrutinib therapy. Apixaban has 27% clearance through the kidneys, and most of it is cleared through hepatic metabolism (75%) [7]. Our patient had normal renal and hepatic function parameters. Given her risk factors for bleeding in the setting of ibrutinib use, as well as the concomitant use of apixaban, we believe together these factors played a role in facilitating the development of hemopericardium in our patient. There are a handful of cases reporting hemorrhagic pericardial effusion in patients with cancer who are on therapy with DOACs and ibrutinib [5,16,17].

Bleeding from ibrutinib use occurs through impaired glycoprotein VI and collagen-mediated platelet aggregations. Bleeding has been shown to be reversed following discontinuation of ibrutinib and/or platelet transfusion [18]. The use of anticoagulants impairs secondary hemostasis; therefore, it is quite obvious that the combined use of ibrutinib along with anticoagulants will impair both primary and secondary hemostasis resulting in an increased risk of bleeding. Combined use of either an antiplatelet agent or an anticoagulant with ibrutinib doubles the risk of bleeding when compared to the use of ibrutinib alone [15].

In cancer patients, there is an additional possible explanation for hemorrhage from DOACs. Cancer patients, in general, have elevated levels of cytokines, interleukin-6 and tumor necrosis factor-alpha which tend to increase following immunotherapy [19]. They alter the pharmacokinetics of drugs by downregulating the expression and enzyme activity of the CYP3A4, the enzyme responsible for the metabolism of DOACs. National Comprehensive Cancer Network guidelines recommend against using DOACs in cancer patients due to the lack of adequate data regarding the safety in this population subgroup [20]. It is essential to be mindful of hemorrhagic complications while placing cancer patients on DOACs. Table 1 demonstrates the number of cases that have thus far been reported along with the demographics of the patients and indications for anticoagulation with DOACs. We found no cases where symptoms presented after one year of DOAC use (Table 1).

**Table 1:** All reported cases of hemorrhagic and non-hemorrhagic pericardial effusions in the setting of DOAC use a-fib: atrial fibrillation, AC: anticoagulation, NA: duration of treatment or indication of use was not mentioned in the article, M: male, F: female, DOAC: direct oral anticoagulant.

Article	Gender	Age	Anticoagulant	Duration of treatment	Indication of AC use
Cinelli et al., 2019 [2]	F	78	Apixaban	7 days	Non-valvular a-fib
Asad et al., 2019 [3]	M	62	Apixaban	8 weeks	Non-valvular a-fib
Khalid et al., 2018 [5]	M	68	Apixaban	8 weeks	Non-valvular a-fib
Sigawy et al., 2015 [11]	F	76	Apixaban	6 weeks	Non-valvular a-fib
Mehta et al., 2019 [12]	F	76	Rivaroxaban	3 weeks	Non-valvular a-fib
Oladiran et al., 2018 [13]	M	87	Rivaroxaban	NA	NA
Nassif et al., 2017 [16]	F	43	Rivaroxaban	2 days	Deep vein thrombosis
Nassif et al., 2017 [16]	M	47	Rivaroxaban	8 weeks	Pulmonary embolism
Nassif et al., 2017 [16]	M	71	Apixaban	2 days	Non-valvular a-fib
Berti et al., 2022 [17]	M	75	Apixaban	NA	Non-valvular a-fib
Dy and Shiltz, 2012 [21]	M	70	Dabigatran	4 months	Non-valvular a-fib
Dy and Shiltz, 2012 [21]	F	77	Dabigatran	3 days	Non-valvular a-fib
Barton et al., 2012 [22]	M	70	Dabigatran	8 weeks	Non-valvular a-fib
Xu and MacIsaac, 2014 [23]	M	75	Rivaroxaban	NA	Non-valvular a-fib
Abdallah et al., 2015 [24]	M	63	Dabigatran	2 weeks	Paroxysmal a-fib
Kızılırmak et al., 2016 [25]	F	66	Dabigatran	12 months	Non-valvular a-fib
Menendez and Michel, 2016 [26]	M	69	Rivaroxaban	3 days	Paroxysmal a-fib
Basnet et al. 2017 [27]	F	56	Rivaroxaban	7 weeks	Deep vein thrombosis
Bastida et al. 2017 [28]	M	77	Apixaban	1 week	Non-valvular a-fib
Gowani et al., 2017 [29]	M	68	Rivaroxaban	6 months	Paroxysmal a-fib
Jelani et al., 2017 [30]	M	87	Rivaroxaban	8 weeks	Non-valvular a-fib
Rhew and Kim, 2017 [31]	M	83	Rivaroxaban	2 months	Non-valvular a-fib
Sablani et al., 2017 [32]	M	70	Apixaban	10 days	Non-valvular a-fib
Sablani et al., 2018 [32]	M	60	Apixaban	15 days	Non-valvular a-fib
Bitar et al., 2020 [33]	F	84	Dabigatran	NA	Non valvular a-fib
Jou-Valencia and Dijkstra, 2020 [34]	M	72	Rivaroxaban	4 days	Pulmonary embolism
Lefas et al., 2020 [35]	M	86	Rivaroxaban	8 days	Non-valvular a-fib
Shastri et al., 2021 [36]	M	84	Rivaroxaban	NA	Non-valvular a-fib
Olagunju et al., 2021 [37]	M	80	Apixaban	2 weeks	Paroxysmal a-fib

## Conclusions

Based on our literature search, hemopericardium appears to be a consistently reported, albeit rare, side effect of DOAC use. In most cases, rivaroxaban was the most likely culprit. This adverse effect also appears to manifest early after initiation of the DOAC with almost all cases presenting within one year of initiation of the anticoagulation. Given that atrial fibrillation is the most common arrhythmia, there has been an increase in the prevalence of anticoagulation use. We aim to highlight hemopericardium as a rare but life-threatening side effect of DOAC use, especially within the first year of therapy and in patients with a history of malignancy or concomitant ibrutinib use. We also encourage that providers offer closer monitoring to patients within the first year of DOAC use and maintain a high index of suspicion for spontaneous hemopericardium. We also urge caution with anticoagulation use, especially in patients with a history of malignancies.
